# Development of a novel recessive genetic male sterility system for hybrid seed production in maize and other cross‐pollinating crops

**DOI:** 10.1111/pbi.12477

**Published:** 2015-10-06

**Authors:** Yongzhong Wu, Tim W. Fox, Mary R. Trimnell, Lijuan Wang, Rui‐ji Xu, A. Mark Cigan, Gary A. Huffman, Carl W. Garnaat, Howard Hershey, Marc C. Albertsen

**Affiliations:** ^1^ DuPont PioneerJohnstonIAUSA; ^2^ Present address: Grand AgriSeeds Technology, Inc.Zijing Information Bldg.Zijing Rd.HaikouHainanChina; ^3^ Present address: Bayer CropScience LP3500 Paramount ParkwayMorrisvilleNC27560USA; ^4^ Present address: Department of ChemistryDes Moines Area Community CollegeBldg. 42006 S. Ankeny Blvd.AnkenyIA50021USA; ^5^ Present address: Monsanto Company800 N. Lindbergh Blvd.St. LouisMO63167USA

**Keywords:** genetic male sterility, hybrid production, transgenic process, nontransgenic progeny, gamete selectivity, Ms45

## Abstract

We have developed a novel hybridization platform that utilizes nuclear male sterility to produce hybrids in maize and other cross‐pollinating crops. A key component of this platform is a process termed Seed Production Technology (SPT). This process incorporates a transgenic SPT maintainer line capable of propagating nontransgenic nuclear male‐sterile lines for use as female parents in hybrid production. The maize SPT maintainer line is a homozygous recessive male sterile transformed with a SPT construct containing (i) a complementary wild‐type male fertility gene to restore fertility, (ii) an α‐amylase gene to disrupt pollination and (iii) a seed colour marker gene. The sporophytic wild‐type allele complements the recessive mutation, enabling the development of pollen grains, all of which carry the recessive allele but with only half carrying the SPT transgenes. Pollen grains with the SPT transgenes exhibit starch depletion resulting from expression of α‐amylase and are unable to germinate. Pollen grains that do not carry the SPT transgenes are nontransgenic and are able to fertilize homozygous mutant plants, resulting in nontransgenic male‐sterile progeny for use as female parents. Because transgenic SPT maintainer seeds express a red fluorescent protein, they can be detected and efficiently separated from seeds that do not contain the SPT transgenes by mechanical colour sorting. The SPT process has the potential to replace current approaches to pollen control in commercial maize hybrid seed production. It also has important applications for other cross‐pollinating crops where it can unlock the potential for greater hybrid productivity through expanding the parental germplasm pool.

## Introduction

Commercial maize is predominantly grown from hybrid seed produced from the cross‐pollination between two genetically distinct inbred parent lines. One inbred line is selected as the pollen donor (male parent) and the other as the pollen recipient (female parent) on which the hybrid seed will develop. For commercial production of maize hybrid seed, male and female inbred parent lines are planted alternately, in adjacent rows, in isolated fields and allowed to open‐pollinate. To produce pure hybrid seed, the male inbred parent must cross‐pollinate the female inbred parent, and the female inbred parent must be prevented from self‐pollinating.

Several methods have been developed to prevent self‐pollination of the female inbred parent during maize hybrid seed production. Maize is a monoecious plant, that is, it has separate male and female flowers on the same plant. Detasseling, the physical removal of the male‐bearing floral structure at the top of the plant by hand or with mechanical cutters or pullers, remains the predominant method employed by industry to ensure that female parent plants in a commercial hybrid production field will only receive pollen from male parent plants. Mechanical detasseling can remove significant amounts of vegetative material resulting in reductions of as much as 40% of the potential inbred seed yield (Wych, 1988).

Cytoplasmic male sterility (CMS) has been employed by hybrid maize producers, but CMS is not effective in all maize germplasm. Effectiveness depends upon the presence or absence of nuclear restoration genes specific for a given cytoplasm, depending upon whether the germplasm will be used as the male parent or the female parent. This restricts the genetic combinations available for use in maize hybrid seed production. Furthermore, CMS can fail to maintain male sterility, particularly under certain environmental conditions, resulting in some level of pollen production by the CMS female parent and subsequent reduced hybrid seed purity and productivity (Denis *et al*., 1993; Weider *et al*., 2009).

For those crops having flowers that are not amenable to manual emasculation, such as rice, wheat and sorghum, commercial quantities of hybrid seed can only be obtained through the use of male‐sterile female parents created from either chemical or genetic manipulations. A number of chemicals (e.g. auxins, anti‐auxins, halogenated aliphatic acids, gibberellins, arsenicals, ethepon) applied as foliar sprays prior to flowering have been investigated as potential gametocides in commercial production of maize hybrid seed (McRae, 1985), but there has been little industry use of chemical sterilants because of factors that impact obtaining complete pollen sterility. These include the impacts of genotype and environment, as well as the logistical challenges of accurate and timely chemical application. Furthermore, some chemical applications reduced seed yields in excess of 20% because of damage to the female flower (Newhouse *et al*., 1996).

One of the first biotechnology‐based approaches to male sterility was proposed by Mariani *et al*. (1990) and included tapetal‐specific expression of a ribonuclease gene, barnase, to cause complete male sterility. Tapetal‐specific expression of a ribonuclease‐inhibitor gene, barstar, restored fertility to hybrid plants (Mariani *et al*., 1992). This technology is linked with the selectable marker phosphinothricin acetyltransferase (PAT) conferring tolerance to the herbicidal active ingredient glufosinate–ammonium. Timely application of herbicide is essential to remove nontransgenic plants, which adds to costs and complexity. Moreover, the hybrid seed produced is a mixture of transgenic and nontransgenic seed and so must comply with global regulatory requirements governing the cultivation and uses of genetically modified (GM) crops. To date, this approach to male sterility has not been adopted in commercial maize hybrid seed production although it is utilized in some hybrid Indian oilseed mustard production (Ray *et al*., 2007).

Most recently, a glyphosate‐mediated biotechnology‐based male sterility system has been developed for hybrid maize production via applications of glyphosate herbicide (Feng *et al*., 2014). Maize plants are transformed with the *Agrobacterium* sp. *CP4‐EPSPS* gene, conferring tolerance to glyphosate, under expression control of a promoter and intron combination that preferentially directs gene expression to the vegetative and female reproductive tissues. This results in limited or no expression in pollen microspores and tapetum cells. During hybrid seed production, two applications of glyphosate, made around the time of tassel development, inhibit pollen formation and make the female inbred parent male sterile. Timely glyphosate application is essential to ensure male sterility and to eliminate or greatly reduce the need for detasseling. The required timeliness of herbicide applications, combined with the need to visually monitor hybrid seed production fields to confirm that female inbred plants have become male sterile and, if necessary, to implement remedial detasseling, may limit the utility of this approach. Additionally, male parents must have a ‘restoring’ glyphosate transgene to ensure pollen production among the hybrid plants if glyphosate is used for weed control.

Nuclear genetic male sterility is a common spontaneous occurrence in flowering plants (Kaul, 1988). For example, there are over 40 genetic male sterility mutations that have been described in maize (Albertsen and Philips, 1981; Bedinger, 1992). The chromosome map positions of nearly all the presently identified male sterility genes are well characterized (Neuffer *et al*., 1997) with the majority of the mutations being recessive. A number of these recessive nuclear genetic male‐sterile mutants could provide an excellent genetic means of emasculation for hybrid seed production in maize as well as in crops such as rice, wheat and sorghum. All plants used as male parents would, by virtue of being male fertile, contain the wild‐type allele and would be ‘restorers’ for a recessive allele‐based male‐sterile mutation. It is, however, impossible to obtain a pure increase of male‐sterile homozygous recessive female inbred parent seed through self‐pollination because the plant is male sterile. The classic Mendelian approach to increasing seed of a homozygous recessive male‐sterile plant is to cross‐pollinate with male‐fertile plants that are heterozygous for the male sterility allele. Progeny from such a cross will segregate 50% male‐sterile (*ms/ms*) and 50% male‐fertile (*Ms/ms*). This approach, however, does not provide a practical way to produce nuclear genetic male‐sterile parent seed for hybrid seed production as male‐sterile seed cannot be separated from male‐fertile seed.

**Figure 1:**
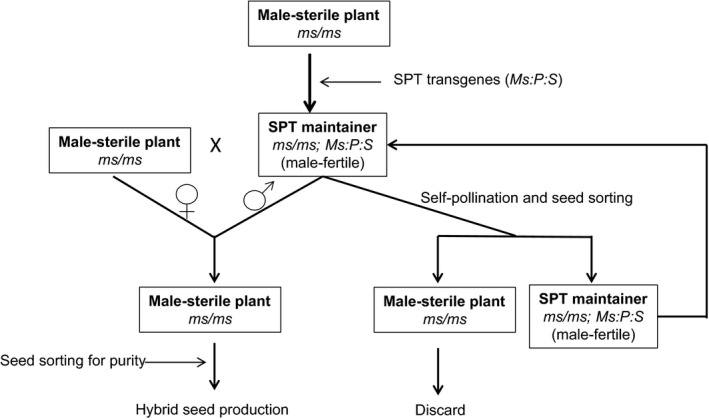
A schematic representation of a system for propagation of recessive genetic male‐sterile (*ms*) plants. *Ms*, male fertility gene; *P*, pollination disruption gene; *S*, seed screenable marker gene; SPT, Seed Production Technology.

We have devised a novel biotechnology‐based process, termed the Seed Production Technology (SPT) process, to propagate seed of homozygous male‐sterile female inbred lines (Figure 1). This publication describes the key steps in the development of the SPT process, the utilization of the technology for maize hybrid seed production, the potential use for other crops and the applicable biotechnology regulatory oversight.

## Results

### Isolation of the *Ms45* male fertility gene

**Figure 2:**
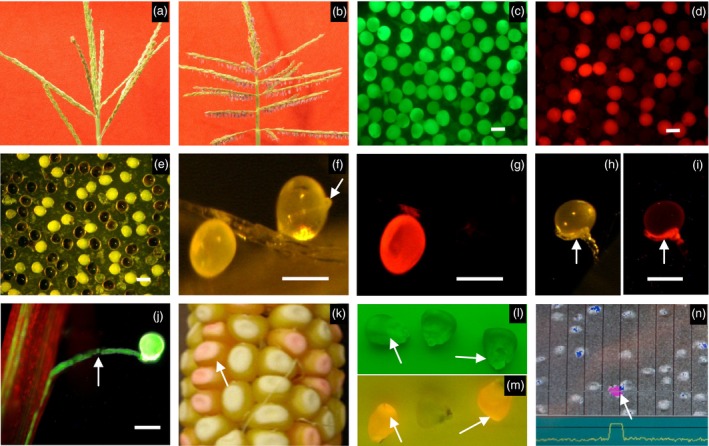
Characterization of transgenic plants. (a) Sterile tassel from *ms45* plant. (b) Fertile tassel from transgenic *ms45* plant containing p*PG* *47::Bt1:zm‐aa1//* p*M* *s5126::Ms45//*p*35S::PAT*. (c–i) Pollen from transgenic plant containing p*PG* *47::Bt1:zm‐aa1//35SEN‐* p*U* *bi::PAT::DsRed‐Express*. (c and d) Mature pollen stained with fluorescein diacetate (c) and also shown under colour filter for red fluorescent proteins (d). (e) Mature pollen sample in (c) stained with potassium iodide. (f–i) *In vivo* pollen germination. (f and g) DsRed‐Express pollen without pollen tube and non‐DsRed‐Express pollen with pollen tube shown under visible light (f) and under colour filter for DsRed‐Express (g). (h and i) Abnormal pollen tube growth from a DsRed‐Express pollen grain shown under visible light (h) and under colour filter for DsRed‐Express (i). (j) Pollen tube growth from a control transgenic plant containing p*U* *bi::YFP*. Arrow in f, and h–j shows pollen tubes. (k–n) Characterization of seed DsRed2 expression and seed sorting. (k) Ear harvested from a transgenic plant bearing p*M* *s5126::Ms45//* p*PG* *47::Bt1:zm–aa1//35SEN‐* p*LTP* *2::DsRed2(Alt1)*. (l and m) Non‐DsRed2 and DsRed2 seeds shown under green filter (l) and under red filter (m). (n) Seeds shown on seed sorting machine as they flow from top to bottom. Arrows in k–n show DsRed2 seeds. Bar = 100 μm.

A maize recessive genetic male‐sterile mutant, *ms45/ms45* (Figure 2a) was identified from a specifically constructed maize Activator (Ac)‐transposon population. Microspore development in this mutant aborts after microspore release from tetrads at about the mid‐vacuolate stage. Although an exine forms in microspores developing in *ms45* mutants, it appears to be defective, possibly due to a defect in sporopollenin biosynthesis (Cigan *et al*., 2001). The wild‐type *Ms45* allele was cloned using an Ac‐transposon‐tagging approach. It was mapped to chromosome 9. A stable *ms45* mutation was found resulting from an imperfect excision of Ac from the *Ms45* allele, leaving an 8‐base pair footprint at the 5′ end of the coding region, resulting in a frame‐shift mutation. The *Ms45* allele shows limited homology to strictosidine synthase genes from *Catharanthus roseus* and *Rauvolfia serpentina* (Kutchan *et al*., 1988; McKnight *et al*., 1990). RNA *in situ* hybridization revealed that *Ms45* is expressed specifically in tapetal cells within the anther (Cigan *et al*., 2001). To demonstrate that the wild‐type *Ms45* allele is capable of complementing the male‐sterile phenotype, the *Ms45* genomic coding region (GenBank: AF360356.1) was inserted behind various anther‐specific promoters (pMs*5126, pMs45 and pMs*BS7) and transformed into *ms45/ms45* plants. It was found that a single transformed copy of the *Ms45* allele, under control of all anther promoters tested, was able to fully restore a male‐fertile phenotype (Cigan *et al*., 2001) (Figure 2b).

### Development of a pollination disruption system

The ultimate goal in designing a system to utilize a nuclear genetic male sterile in commercial hybrid production is to generate all male‐sterile progeny during parent increase. One approach is to prevent inheritance of the *Ms45* wild‐type allele. To this end, a maize α‐amylase gene, *zm‐aa1* (gi|293334594|ref|NM_001176140.1), encoding a putative α‐amylase family protein (LOC1000383492), was evaluated for its ability to interfere with the pollination process. Starch biosynthesis occurs during the final phases of pollen maturation and is critical to providing an energy source for pollen germination, pollen tube growth and ultimately for successful fertilization. Our hypothesis was that disrupting starch accumulation during pollen maturation would deprive pollen carrying this transgene of the necessary energy source required for achieving fertilization. An α‐amylase gene was chosen to test this hypothesis because these enzymes hydrolyse α bonds of large, α‐linked polysaccharides, including starch. The *zm‐aa1* α‐amylase gene from maize was isolated from a cDNA library made from developing seeds. Because starch accumulation in pollen occurs in amyloplasts (Pacini, 1996), a native signal peptide identified in the α‐amylase gene was replaced by the amyloplast‐targeting signal peptide from the maize brittle‐1 gene (*Bt1*) (Sullivan *et al*., 1991). A late pollen‐specific promoter from a maize polygalacturonase gene, PG47 (Allen and Lonsdale, 1993), was used to drive expression of this α‐amylase gene. The PG47 promoter is active soon after the first microspore mitosis, and its activity continues until pollen tube growth, which covers the time period of starch accumulation in maize pollen. The PG47 promoter is not expressed in any other tissues of the maize plant (data not shown).

To examine the effect of α‐amylase expression on pollen fertility, a construct (Table 1) containing p*PG47::Bt1:zm‐aa1* linked to a variant of the *Discosoma* sp. red fluorescent protein gene (*DsRed–Express*) under a constitutive maize ubiquitin (Ubi) promoter (Matz *et al*.,^1999^) was made and transformed into maize plants (*n* = 10). Pollen grains at different development stages were collected from transgenic plants and stained for viability with fluorescein diacetate (FDA) (Figure 2c) and with potassium iodide (I_2_KI) (Figure 2e) to detect starch accumulation. As shown in Figure 2, most mature pollen grains from transgenic plants generated by insertion of the construct (PHP26689) showed a normal pollen phenotype and fluorochrome reaction when stained with FDA (Figure 2c), suggesting that the transgenic pollen is viable. About half of the mature pollen grains showed red fluorescence for DsRed‐Express protein (Figure 2d) and, FDA positive staining notwithstanding, did not contain normal starch after being stained with I_2_KI, whereas the nonred fluorescent pollen grains stained strongly with I_2_KI (compare Figure 2d,e). This experiment definitively links the absence of detectable starch in pollen with the presence of p*PG47::Bt1:zm–aa1*.

**Table 1:** Constructs tested for development of SPT maintainer line

Construct name	Promoter‐gene combination
PHP20784	p*PG47::Bt1:zm‐aa1//*p*Ms5126::Ms45//*p*35S::PAT*
PHP21478	p*PG47::Bt1:zm‐aa1//*p*Ms5126::Ms45//*p*Ubi::PAT*
PHP22625	p*PG47::Bt1:zm‐aa1//*p*Ms45::Ms45//*p*LTP2::DsRed2(Alt1)* *+ *p*35S::BAR* a
PHP24109	p*PG47::Bt1:zm‐aa1//*p*Ms45::Ms45//*p*LTP2::DsRed2(Alt1)*
PHP24418	p*PG47::Bt1:zm‐aa1//*p*Ms5126::Ms45//*p*LTP2::DsRed2(Alt1)*
PHP24485	p*PG47::Bt1:zm‐aa1//*p*Ms5126::Ms45//35SEN‐*p*LTP2::DsRed2(Alt1)*
PHP24490	p*PG47::Bt1:zm‐aa1//*p*Ms45::Ms45//35SEN‐*p*LTP2::DsRed2(Alt1)*
PHP24593	p*Ms45::Ms45//*p*PG47::Bt1:zm‐aa1//35SEN‐*p*LTP2::DsRed2(Alt1)*
PHP24596	p*Ms5126::Ms45//*p*PG47::Bt1:zm‐aa1(rev)//35SEN‐*p*LTP2::DsRed2(Alt1)*
PHP24597	p*Ms5126::Ms45//*p*PG47::Bt1:zm‐aa1//35SEN‐*p*LTP2::DsRed2(Alt1)*
PHP24612	p*Ms45::Ms45//*p*PG47::zm‐aa1(rev)//35SEN‐*p*LTP2::DsRed2(Alt1)*
PHP26689	p*PG47::Bt1:zm‐aa1//35SEN‐*p*Ubi::PAT::DsRed‐Express*
PHP29331	p*PG47::zm‐aa1//*p*Ms5126::Ms45//35SEN‐*p*LTP2::DsRed2(Alt1)*

*Ms45*, maize fertility restorer allele; *zm‐aa1*, α‐amylase gene; PAT, herbicide resistance gene; *DsRed2(Alt1)*, red fluorescent gene; *DsRed‐Express*, red fluorescent gene; 35SEN, cauliflower mosaic virus 35S enhancer; pUbi, ubiquitin promoter; p35S, cauliflower mosaic virus 35S promoter; Bt1, Brittle‐1 transit peptide; BAR, herbicide resistance gene; pMs45, Ms45 gene promoter; pLTP2, lipid transfer protein‐2 gene promoter; pMs5126, Ms*5126 gene promoter; pPG47, polygalacturonase gene promoter; *zm‐aa1(rev)* indicates different orientation of the *zm‐aa1* cassette compared to plasmid with the same promoter‐gene combinations.

a Two T‐DNAs.

To examine the germination potential of the transgenic pollen described above, pollen grains were collected for *in vivo* germination tests. About 70% of the red fluorescent pollen grains (*n* = 170) and 50% of the nonred fluorescent pollen (*n* = 150) did not re‐hydrate, an indication that they were unlikely to have germinated. Maize pollen can be recalcitrant to *in vivo* germination, accounting for the significant lack of nonred fluorescent pollen germination. Some red fluorescent pollen grains did re‐hydrate but were unable to germinate (compare Figure 2f, g). Very few red fluorescent pollen grains germinated; only 0~2 germinating pollen grains were observed per pollination. These red fluorescent pollen grains produced abnormal rudimentary pollen tubes (Figure 2h, i) that were not equivalent to pollen tubes emerging from fluorescing transgenic pollen grains transformed with a maize ubiquitin promoter driving a yellow fluorescent protein gene that were developed as a comparative control (Figure 2j). The germination rate of red fluorescent pollen grains was not determined due to the difficulty in counting the actual number of pollen grains applied to the silks.

To determine whether the α‐amylase fusion gene could prevent transgene transmission through pollen, a construct (Table 1) was made containing the α‐amylase gene linked to the *Ms45* fertility gene and to the PAT herbicide resistance gene and transformed into homozygous *ms45* plants. All transformants (*n* = 18) showed a normal fertile tassel phenotype, indicating that the cloned wild‐type *Ms45* allele in this construct complemented the male‐sterile mutation (Figure 2b). The T_0_ plants were used as males to outcross onto nontransgenic plants; they also were crossed as females with a nontransgenic pollen source. Embryos (18 days after pollination) from these crosses were plated onto a herbicide selection medium consisting of 3 mg/L bialaphos. No embryos (*n* = 1800) from male T_0_ crosses germinated while embryos (*n* = 1800) harvested from female T_0_ crosses showed ~50% of the progeny resistant to glufosinate herbicide (data not shown). These results demonstrate that the linked transgenic herbicide resistance gene is not being transferred to progeny through pollen, but that it transmits normally through egg cells as predicted by the specificity of the PG47 promoter.

To test whether the linked transgenic male fertility gene (pMs*5126::Ms45) responsible for the fertility of the SPT maintainer plant would be transmitted through the pollen, pollen grains from transgenic SPT maintainer plants were crossed onto nontransgenic homozygous *ms45* plants. All but one progeny (*n* = 3356) exhibited the male‐sterile phenotype, indicating that these transgenic plants are capable of maintaining the homozygous condition of the *ms45* allele to achieve male sterility while not transmitting the *Ms45* male fertility transgene. Transgene transmission through pollen was then tested on a larger scale. Progenies (*n* = 95 866) were produced using transgenic plants as male parents to pollinate nontransgenic plants. The progenies were sprayed with glufosinate herbicide, and 25 resistant plants were found. Southern blots confirmed that these plants contained the three intact transgenes (data not shown). Thus, the transgene transmission rate through pollen for the events generated from this construct was about 0.026%.

### Development of a screenable colour marker for identifying transgenic seed

To facilitate direct identification and removal of transgenic seeds and further enhance the robustness of the SPT process, the herbicide resistance gene was replaced with a screenable seed marker gene [*DsRed2(Alt1)*] encoding the DsRed2 protein, a version of the *Discosoma* sp. red fluorescent protein (Table 1). Expression of this gene was driven by a promoter from a barley lipid transfer protein (LTP2) gene, which is preferentially expressed in the endosperm (Kalla *et al*., 1994). Seeds that were transformed with constructs containing the *DsRed2(Alt1)* gene could be visually distinguished from nontransgenic yellow seeds by their pink colour (Figure 2k) and by their strong red fluorescence under appropriate illumination. This demonstrated that the *DsRed2(Alt1)* fusion gene could serve as a visible, screenable marker to identify transgenic seeds.

### Selection of the SPT maintainer line

An effective SPT maintainer line must reliably propagate the *ms45/ms45* genotype without transmitting SPT‐related transgenes to progeny during male‐sterile parent increase. To effectively combine fertility restoration, pollination disruption and transgenic seed identification functions in a single SPT maintainer line, we extensively tested various SPT constructs containing the α‐amylase transgene in different combinations and orientations with the *Ms45* male fertility allele and the *DsRed2(Alt1)* gene (Table 1). These constructs were transformed into homozygous *ms45* male‐sterile plants. Transformants (*n* = 20 per construct) were evaluated for whole plant morphology including male fertility, *DsRed2(Alt1)* gene expression and transgene transmission through pollen and egg cells. All transformants showed a fertile tassel phenotype, and about 50% of their seeds were pink due to expression of *DsRed2(Alt1)* (Figure 2k). Progenies grown from pink seeds from selfed SPT maintainer lines exhibited tassel fertility whereas plants grown from yellow seeds from selfed SPT maintainer lines were completely male sterile (Table 2). This indicates that the male fertility phenotype is linked with the presence of the transgenic construct, and that SPT maintainer transgenic plants can be maintained by selecting pink seeds expressing the DsRed2 protein.

**Table 2:** Male fertility of self‐pollinated progenies from transgenic SPT maintainer plants

Number of plants with observed phenotype/total number of plants (%)			
Promoter‐gene combination and transformant	Fully fertile	Partially fertile	Completely sterile
Fluorescent seeds			
p*PG47::Bt1:zm‐aa1//*p*Ms5126::Ms45//35SEN‐*p*LTP2::DsRed2(Alt1)* Event E6499.105.6.7	28/28 (0)	0/28 (0)	0/28 (0)
p*Ms5126::Ms45//*p*PG47::Bt1:zm‐aa1//35SEN‐*p*LTP2::DsRed2(Alt1)* Event DP‐32138‐1	188/194 (96.9)	6/194 (3.1)	0/194 (0)
p*Ms45::Ms45//*p*PG47::Bt1:zm‐aa1//35SEN‐*p*LTP2::DsRed2(Alt1)* Event E6611.22.8.2	19/21 (90.5)	2/21 (9.5)	0/21 (0)
p*PG47::Bt1:zm‐aa1//*p*Ms45::Ms45//*p*LTP2::DsRed2(Alt1)* Event E6499.75.6.3	31/37 (83.8)	6/37 (16.2)	0/37 (0)
Nonfluorescent seeds			
Nontransgenic progeny of event E6499.105.6.7	0/14 (0)	0/14 (0)	14/14 (100)
Nontransgenic progeny of event DP‐32138‐1	0/35 (0)	0/35 (0)	35/35 (100)
Nontransgenic progeny of event E6611.22.8.2	0/13 (0)	0/13 (0)	13/13 (100)
Nontransgenic progeny of event E6499.75.6.3	0/31 (0)	0/31 (0)	31/31 (100)

Self‐pollinated seeds segregate for *DsRed2(Alt1)* gene. *Ms45*, maize fertility restorer allele; *zm‐aa1*, α‐amylase gene; *DsRed2(Alt1)*, red fluorescent gene; 35SEN, cauliflower mosaic virus 35S enhancer; Bt1, Brittle‐1 transit peptide; pMs45, Ms45 gene promoter; pLTP2, lipid transfer protein‐2 gene promoter; pMs5126, Ms*5126 gene promoter; pPG47, polygalacturonase gene promoter.

To examine the stability of transgenes in prospective SPT maintainer lines, genomic DNA from T_0_, T_1_, T_2_, T_3_ and T_4_ plants was used for Southern blot analyses to assess the integration and structural fidelity of the transgenes. No changes in hybridization patterns were observed with at least three different restriction enzyme digestions of each of the three SPT transgenes, indicating that the transgenes are stable over multiple generations (data not shown). To test transgene transmission through pollen, transgenic SPT maintainer plants from two constructs (differing by the promoter driving *Ms45*; Table 3) were used to pollinate nontransgenic plants. Ears were harvested from the nontransgenic female parent plants and examined under visible light for the presence of pink seeds expressing the DsRed2 protein. These tests were carried out over five generations and in five different genetic backgrounds (Table 3). The transgene transmission rate through pollen (based on seeds expressing DsRed2) varied with different constructs and transformants (three of which are shown in Table 3). Transformant DP‐32138‐1 (Table 3) showed the lowest transgene transmission rate that was maintained across generations and in different inbred backgrounds. This suggests that over 99.99% of the transgenic pollen grains from this transgenic event were unable to achieve fertilization. Subsequently, transformant DP‐32138‐1 from construct PHP24597 (Table 1) was selected as the SPT maintainer line for use in maize male‐sterile parent seed increase. Detailed information about the DP‐32138‐1 SPT maintainer line can be found in Weber *et al*. (2009).

**Table 3:** Examples of transgene transmission through pollen of different SPT maintainer transformants

Generation/Background^a^	Observed number of *DsRed2(Alt1)* seeds/total number of seeds produced by nontransgenic plants pollinated by transgenic SPT plants (%)		
	Promoter‐gene combination		
	p*Ms5126::Ms45//* p*PG47::Bt1:zm‐aa1// 35SEN‐*p*LTP2::DsRed2(Alt1)*	p*PG47::Bt1:zm‐aa1//* p*Ms45::Ms45//* p*LTP2::DsRed2(Alt1)*	p*PG47::Bt1:zm‐aa1//* p*Ms45::Ms45//* p*LTP2::DsRed2(Alt1)*
	Transformant		
	Event DP‐32138‐1	Event E6209.109.1.4	Event E6499.75.6.3
Generation			
T_0_	0/362 (0)	0/171 (0)	0/926 (0)
T_1_	0/19 012 (0)	0/9354 (0)	4/9698 (0.041)
T_2_	2/86 126 (0.002)	3/16 263 (0.018)	18/91 508 (0.02)
T_3_	4/213 689 (0.002)	0/6916 (0)	61/143 828 (0.042)
T_4_	3/437 298 (0.0007)	9/57 213 (0.016)	468/144 825 (0.323)
Background			
A	0/78 354 (0)	N/A^b^	N/A
B	0/77 108 (0)	6/71 455 (0.008)	356/69 659 (0.511)
C	0/38 421 (0)	7/13 658 (0.051)	100/19 295 (0.518)
D	N/A	N/A	12/55 871 (0.021)
E	N/A	0/58 972 (0)	N/A

T_0_, T_1_, T_2_, T_3_ and T_4_ are primary transformants, first, second, third and fourth generations, respectively. *Ms45*, maize fertility restorer allele; *zm‐aa1*, α‐amylase gene; *DsRed2(Alt1)*, red fluorescent gene; 35SEN, cauliflower mosaic virus 35S enhancer; Bt1, Brittle‐1 transit peptide; pMs45, Ms45 gene promoter; pLTP2, lipid transfer protein‐2 gene promoter; pMs5126, Ms*5126 gene promoter; pPG47, polygalacturonase gene promoter.

a Key genetic inbred background.

b Data not available.

### Confirmation that progeny seed do not inherit the SPT transgenes

We conducted molecular analyses to confirm the absence of the SPT transgenes in male‐sterile progeny produced using the DP‐32138‐1 SPT maintainer line. The SPT maintainer line was used as a pollen donor to cross with a nontransgenic male‐sterile (*ms45*) maize line. The progeny seed from the male‐sterile line was hand‐harvested and passed twice through a high‐speed optical colour sorter to separate any red fluorescent SPT transgenic seed from nonfluorescent, nontransgenic progeny seed. Fifteen thousand nonfluorescent (yellow) seeds were randomly selected from the sorted population and planted in the greenhouse. Two leaf samples were collected from each seedling, and genomic DNA was extracted from each of the samples. Each genomic DNA sample was analysed using real‐time PCR assays for the three SPT transgenes present in the SPT construct: *Ms45*,* zm‐aa1* and *DsRed2(Alt1)*. The three gene assays on the samples were run by independent PCR reactions. Nearly every plant was analysed in duplicate. In total, all 15 000 plants were analysed at least once by real‐time PCR (Table S1).

After both rounds of PCR analyses, 14 998 plants tested negative for all three PCR assays, confirming the absence of the SPT transgenes (Table S1). Positive and negative control samples gave the expected results during each run. One progeny maize plant was negative for the *Ms45* and *DsRed2* PCR assays, but it tested positive in both rounds of PCR analysis for *zm‐aa1*. Subsequent molecular analysis showed that the unexpected genotype was the result of contamination by a rDNA construct from a different source T‐DNA plasmid containing a similar *zm‐aa1* cassette. A second plant that had been negative for all assays in the first round of PCR analysis tested positive for the *DsRed2(Alt1)* gene and negative for the *zm‐aa1* and *Ms45* genes in the second round of PCR analysis. The result was attributed to contamination of the second sample by a different source of *DsRed2*. Thus, based on the two independent rounds of PCR analysis, we can conclude that none of the 15 000 male‐sterile progeny plants originating from the DP‐32138‐1 SPT maintainer line used as a pollinator contained the SPT transgenes. This demonstrates the extremely high efficacy of the SPT genetic process in preventing the transfer of the SPT transgenes to the male‐sterile female parents being increased. Furthermore, this experiment demonstrates that large‐scale automated seed sorting can be employed to further help ensure the purity of the nontransgenic male‐sterile inbred parent seed being increased and subsequently used for commercial scale maize hybrid seed production. Because the SPT transgenes are transmitted only through the kernel, the SPT maintainer line can be readily propagated by self‐pollination followed by sorting the resulting seed mix and retaining the red fluorescent seeds.

## Discussion

We have described the steps leading to the development of a novel biotechnology‐based process that can efficiently propagate genetic male‐sterile plants for use as female inbred parents in maize hybrid seed production, thereby eliminating the need to detassel plants. The SPT process described utilizes a homozygous recessive transgenic SPT maintainer line, DP‐32138‐1, containing (i) a cloned male fertility gene (*Ms45*) that functions sporophytically to restore male fertility, (ii) a pollination disruption gene (*zm–aa1*) expressing an α‐amylase that functions only in the male gametes (pollen) that inherit it and (iii) a screenable fluorescent colour marker gene [*DsRed2*(*Alt1*)] that can be used to identify and to facilitate purification of the transgenic SPT maintainer seed.

The SPT construct is integrated into the maintainer line in a hemizygous state. Consequently, 50% of the pollen grains produced are capable of fertilization, as they lack the SPT transgenes, and 50% are depleted of starch by α‐amylase activity and incapable of fertilization because of the presence of the SPT transgenes. Pollen inheritance of the SPT transgenes through the SPT maintainer line is effectively blocked by the pollen‐specific expression of the *zm‐aa1* gene. Fertility of the SPT maintainer line is maintained by a single copy of the *Ms45* allele in the SPT construct. Because the SPT transgenes can only be inherited through the female parent, self‐pollination of the SPT maintainer line will maintain the hemizygous condition of the SPT transgenes. The resulting mixture of 50% yellow seeds, which do not contain the SPT transgenes, and 50% fluorescent seeds that do contain the SPT transgenes can be separated by optical colour sorting, in which case the fluorescent SPT maintainer seed is retained (Figure 1). The SPT maintainer line is used to increase the seed of a nontransgenic male‐sterile line without the SPT transgenes being inherited.

Introgression of the *ms45* allele and the SPT transgenes into female lines in a breeding programme is straightforward. Both the *ms45* allele and the SPT transgenes can be introgressed at the same time. The SPT construct is easily selected each generation by selecting for the pink/fluorescent kernels. The *ms45* allele can be selected after PCR analysis of either the 8‐base pair footprint characteristic of that allele or by a specific SNP assay for that allele.

Experimental analyses of the SPT genetic system confirmed its potential to be deployed on a commercial scale for maize hybrid seed production. To help ensure that no inbred male‐sterile female parent seed contains the SPT rDNA construct, the seed is passed through a high‐speed optical colour sorter to remove seeds exhibiting DsRed2 protein fluorescence before being planted in maize hybrid seed production fields (Figure 1).

The SPT process requires no detasseling, works across all maize germplasm and increases hybrid seed production yields and hybrid seed purity. In contrast with other male sterility systems, the SPT process allows for the reliable restoration of fertility in the hybrid plants grown in commercial maize fields. Because this version of the process utilizes a recessive, genetic nuclear male‐sterile mutant in the female inbred parent, crosses with any male‐fertile male inbred parent will restore male fertility to their F1 hybrid progeny. Another key desirable factor is that the process is limited to the parent seed increase phase of hybrid production. This phase involves acreage that is approximately two orders of magnitude less than the acreage involved during the hybrid production phase.

**Figure 3:**
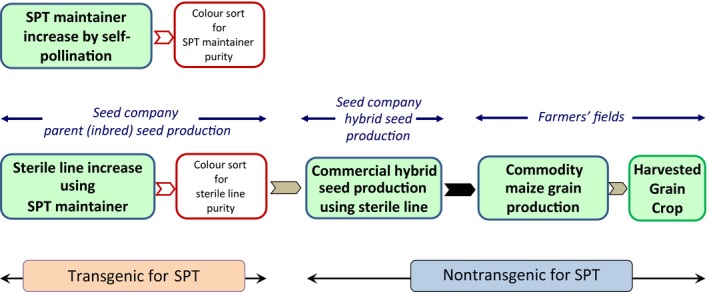
Schematic representation of the Seed Production Technology (SPT) process with subsequent commercial hybrid seed and commodity maize grain production. The steps involving the cultivation of the transgenic SPT maintainer line are under control of the seed producer. Inbred parent sterile line, hybrid seeds planted by the farmer and the harvested grain crop are nontransgenic for SPT. Maize grain that enters the food and animal feed supply is three generations removed from the point at which the SPT maintainer line was used in parent seed production.

The SPT process, combining the SPT genetic male sterility seed increase system with high‐speed optical seed colour sorting, has been successfully deployed in US maize hybrid seed production operations since 2012 (Figure 3). The SPT process has the potential to dramatically impact hybrid maize seed production as it eliminates a need for mechanical and/or hand detasseling. Soil compaction and fuel inputs also will be reduced by the elimination of operating mechanical detasseling equipment in the hybrid production fields. Furthermore, the number of temporary workers required to manually detassel hybrid seed production fields will be greatly reduced. Hybrid seed production yields on the female parent lines are expected to increase as mechanical detasseling often results in removal of several leaves, which negatively impacts plant photosynthetic capacity and can lead to secondary disease susceptibility.

Unlike other biotechnology approaches to male sterility, the maize inbred parent lines produced using the SPT process do not inherit SPT rDNA from the SPT maintainer line and are, therefore, nontransgenic. Furthermore, both the commercial maize hybrid seed produced using male‐sterile female inbred parents resulting from the SPT process and the resulting commodity maize grain harvested from these hybrid plants do not contain the SPT rDNA, and are, therefore, nontransgenic for SPT rDNA. Acknowledgement of the nontransgenic status (for SPT rDNA) of progeny produced by the SPT process is supported, for example, by regulatory agencies in the USA (USDA‐APHIS, 2011a), Australia (FSANZ, 2012), and Japan (Japan MHLW, 2013). These acknowledgements have important regulatory and commercial implications for hybrid seed products produced using the SPT process. Therefore, in the absence of other transgenes or rDNA, hybrid maize and commodity grain produced from the SPT process are non‐GM and subject only to those regulations applicable to conventional non‐GM maize.

The SPT maintainer line itself is GM and is subject to biotechnology regulatory oversight proportionate for its limited and specific cultivation during company‐controlled inbred maize parent seed production. Even if the SPT process were to be deployed by the entire US seed production industry, the SPT maintainer line is anticipated to only be planted on <0.023% of the total acreage of the US commercial maize crop. The SPT maintainer line, DP‐32138‐1, has been deregulated by the US Department of Agriculture Animal and Plant Health Inspection Service (USDA‐APHIS, 2011b), which allows planting of the SPT maintainer line for seed production.

The SPT process also has potentially important applications for hybrid seed production in other major crops including rice, sorghum and wheat, which have flowers that are not amenable to manual emasculation. Enhancing the ability to produce hybrids is anticipated to significantly improve grain productivity gains in these crops. For example, varietal rice, which makes up approximately 90% of today's commercial production, will be challenged to meet a growing food demand. The development of rice hybrids could help address this demand, but today's methods for creating higher yielding rice hybrids are limited by a combination of genetic and environmental factors (Virmani *et al*., 2003). Currently used hybridization platforms rely upon systems that limit the range of germplasm available for developing the most productive hybrids because of either the genetics or the specific environmental requirements for that particular platform. We estimate that less than 30% of the rice germplasm is available to plant breeders due to these limitations, which has slowed the adoption of hybrid rice where grain quality standards have been difficult to meet. The SPT process would enable breeders to make breeding choices strictly on the suitability of the germplasm to be used as parents in making a hybrid. This process represents an important advancement in capturing the benefits of hybridization to feed growing populations across a range of hybridizing crop plants.

## Experimental procedures

### Maize transformation and molecular analyses of transformants

Male‐sterile transformation‐amenable maize lines, which contain the *ms45′‐9301* allele (cloning described in Data S1), were developed by backcrossing with pollen from a transformable maize genotype. Immature embryos that segregated for the *ms45′‐9301* allele (1 : 1 or 3 : 1) were used for transformation. This enabled transformation directly into a homozygous *ms45′‐9301* background to test the genetic complementation of the *ms45* mutation with the putative *Ms45* allele in the transformed plants (T_0_). *Agrobacterium*‐mediated transformation, DNA isolation, PCR analyses, Southern and Western blot hybridization were carried out as previously described (Cigan *et al*., 2001).

### Histochemical analysis, pollen *in vivo* germination and microscopy

Anthers containing microspores or pollen grains at different developmental stages were gently squashed on a glass slide using a fine forceps in a drop of staining solution. Viability examination was carried out using FDA (Heslop‐Harrison and Heslop‐Harrison, 1970). Staining for starch with I_2_KI solution was performed according to Nelson (1968). For transgenic plants bearing α‐amylase and DsRed‐Express (Clontech, Mountain View, CA), pollen samples were first stained for viability with FDA, then examined for red fluorescence. The samples were then stained for starch with I_2_KI without disturbing the sampl es. For *in vivo* pollen germination, freshly shed pollen grains were pollinated onto ears with silk 5 cm long. Silks were then collected at 1, 2, 3 and 4 h after pollination and examined under a light microscope.

### Seed sorting and sorter specifications

A ScanMasterII 200 high‐volume colour sorter (Satake‐USA, Houston, TX) was used to sort DsRed2 seeds from non‐DsRed2 seeds (Weber *et al*., 2009). This sorter has 20 narrow parallel channels through which the seeds run rapidly. Single seeds are viewed by both front and rear charge coupled device (CCD) cameras. The sorter was equipped with red and green filters that maximize the colour difference between DsRed2 and non‐DsRed2 expressing seeds when examined under specific illumination (Figure 2l,m). The seeds expressing DsRed2 protein can be clearly detected (Figure 2n) and removed. The background level can be adjusted by settings with different parameters including light trip, feed rate and reject rate. In most cases, it only required two sorting passes to reach 100% purity of non‐DsRed2 yellow seeds. To confirm that these settings were optimal for detecting and separating occasional DsRed2 seeds, 24 pink DsRed2 seeds were mixed into 48 000 yellow seeds and the sorting was repeated twice (i.e. two passes). In four tests, all 24 DsRed2 seeds were rejected after the first sorting pass. Additional data regarding the accuracy of the colour sorter to detect and separate fluorescing transgenic SPT maintainer seed from male‐sterile progeny seed can be found in Weber *et al*. (2009).

### Real‐time PCR of male‐sterile progeny plants

Real‐time PCR was performed utilizing an ABI PRISM^®^ 7900HT Sequence Detection System (Applied Biosystems, Inc., Foster City, CA). TaqMan^®^ probe and primer sets were designed to detect three different target sequences within the T‐DNA: the Ms*5126 promoter/*Ms45* junction (*Ms45* assay), the *zm–aa1*/*In2‐1* terminator junction (*zm‐aa1* assay), or within the *DsRed2(Alt1)* gene [*DsRed2(Alt1*) assay]. The target sequences were selected so that they would be specific to their respective transgenic cassettes and would not amplify sequences within the native maize genome. Each of the 15 000 test plants was analysed with all three PCR assays. In addition, a TaqMan^®^ probe and primer set for a maize alcohol dehydrogenase gene (ADH) was duplexed with each target PCR reaction to serve as a reference endogenous gene to confirm the presence of amplifiable DNA in each reaction. The extracted DNA was assayed using optimized and validated primer and probe concentrations in Extract–N–Amp^™^ PCR reaction mix (Sigma‐Aldrich, St. Louis, MO) containing ROX^™^ Passive Reference Dye (Sigma‐Aldrich). Positive and negative control DNA samples were added to each plate run with the cassette PCR assays. After initial incubation at 95 °C for 3 min, 40 cycles were conducted as follows: 95 °C for 15 s and 60 °C for 1 min. Positive or negative determination for each target sequence assay was based on comparison of the *C*_T_ (threshold cycle) of the target PCR assay to that of the ADH endogenous reference assay. The interpretation of the results consisted of real‐time PCR determination of qualitative positive/negative calls.

## Conflict of interest

YW, TWF, MRT, GAH, AMC, CWG, HH and MCA are inventors on awarded patents and pending applications on this work and are current or former employees of DuPont Pioneer who owns the patents and pending patent applications.

## Supporting information


**Table S1** PCR analysis for *Ms45*,* zm‐aa1*, and *DsRed2(Alt1)* transgenes of the male‐sterile progeny plants from the DP‐32138‐1 SPT maintainer line.
**Data S1** Experimental procedures describing cloning of the *Ms45* gene.
