# Supplementary material for: Development of a novel recessive genetic male sterility system for hybrid seed production in maize and other cross‐pollinating crops
Source: Plant Biotechnol J. 2015 Oct 6;14(3):1046–54. doi: 10.1111/pbi.12477 (PMC5057354; doi:10.1111/pbi.12477)
Supplement: Supplementary file 1 — Table S1 PCR analysis for Ms45, zm‐aa1, and DsRed2(Alt1) transgenes of the male‐sterile progeny plants from the DP‐32138‐1 SPT maintainer line. Data S1 Experimental procedures describing cloning of the Ms45 gene. [file PBI-14-1046-s001.docx]

**Supporting Information**

**Table S1** PCR analysis for *Ms45*, *zm-aa1*, and *DsRed2(Alt1)* transgenes of the male-sterile progeny plants from the DP-32138-1 SPT maintainer line.

| **Analysis round** | **Total samples analyzed** | **Negative for all PCR assays** | **Insufficient DNA^†^** | **Positive for *zm-aa1* assay^‡^** | **Positive for *DsRed2(Alt1)* assay^§^** |
| --- | --- | --- | --- | --- | --- |
| 1 | 15,000 | 14,989 | 10 | 1 | 0 |
| 2 | 15,000 | 14,973 | 25 | 1 | 1 |

^†^Insufficient DNA for amplification in either the endogenous control or transgene assays.

^‡^Negative for *Ms45* and *DsRed2(Alt1)*

^§^Negative for *Ms45* and *zm-aa1*

**Data S1**

**Cloning of the *Ms45* gene**

Transposon tagging experiments were initiated utilizing a maize genetic stock containing an Activator (Ac) element at the *P* locus (*P-vv*) in maize crossed with the white inbred line 4Co63. Selections were made for kernels where Ac had transposed out of the *P* locus (indicated by a red-striped pericarp over at least half of the embryo). The resultant plants were selfed to screen for Ac-induced mutations. In total, 972 families were screened, and163 families were found to segregate for male sterility (data not shown). Co-segregation analyses of Ac and male sterility were conducted both through field-genetics screening and through molecular analysis. Field-genetics screening was accomplished by tracking the active Ac element with a *Ds*-containing stock (W22 *r-sc:m3*). This stock is characterized by a *Ds* element integrated into the *R*-gene, in addition to having all the alleles required for kernel anthocyanin development. Because *Ds* is a non-autonomous element, the *Ds*-interrupted *R*-gene represents a stable mutation and kernels from this stock are yellow rather than purple. Kernels develop purple spots when this stock is crossed with lines containing an active Ac element because the Ac element mediates *Ds* transposition, restoring *R*-gene function and subsequently, anthocyanin production. Anthocyanin production was used to correlate the presence of Ac with male sterility.

One family, TRHN90-40, was found to segregate 13 male-fertile plants to four male-sterile plants. To determine if the Ac element was linked to male sterility, a testcross using the segregating male-sterile family TRHN90-40 as a female parent was made with the W22 *r-sc:m3* tester line. Thirty-three ears from this cross were screened for the Ac-induced excision of *Ds* from the *R* lo­cus, represented by purple‑spotted kernels. If Ac had transposed into, and disrupted a gene responsible for male fertility, then every kernel on ears from male-sterile plants should have Ac and be purple-spotted after crossing with the *Ds*‑tester line. Of the male-fertile plants, two-thirds are expected to be heterozygous for Ac and to segregate yellow kernels and purple-spotted kernels. The remaining male-fertile plants are not expected to carry Ac and will have ears with all yellow kernels. Eight of the 33 plants were male sterile and all eight had ears homozygous for active Ac (all kernels were purple spotted), 16 plants were male fertile and heterozygous for active Ac (50% yellow and 50% purple-spotted kernels), and nine plants were male fertile and did not have Ac present (all yellow kernels) (data not shown). These results support the expected ratio of 1:2:1 (*X*2 = 0.15, p > 0.90) for Ac being responsible for the recessive mutation.

The co-segregation of Ac with male sterility was confirmed molecularly using an Ac internal *Hin*dIII fragment to probe *Pvu*II-restricted DNA isolated from F_2_ progenies. A 6.0-kb *Pst*I fragment was identified that hybridized to an Ac internal *Hind*III fragment and that co-segregated with all plants shown to have an active Ac. *Pst*I-digested DNA with a molecular weight between 5.0 and 7.0 kb was isolated and used as the template in an inverse polymerase chain reaction (IPCR). A single 1.3-kb amplified product was obtained in close agreement with the predicted amplicon size. A DNA blot containing *Pst*I-digested DNA from the segregating F_2_ Ac male-sterile family was hybridized with the entire IPCR product. The blot showed that all fertile plants are either homozygous for a 1.3-kb band or heterozygous for 1.3-kb and 6.0-kb bands. Male-sterile plants were shown to be homozygous for the 6.0-kb *Pst*I fragment containing the Ac element, indicating genetic linkage of the IPCR product with the mutant phenotype.

The IPCR product was used to screen a cDNA library constructed from maize tassel mRNA. A 1.4‑kb clone was isolated. An internal probe was made from the cDNA by digesting with *Pst*I and was used to hybridize to the *Pst*I co-segregation blot. This probe was found to hybridize as a single copy gene to the same genomic fragments as the IPCR probe, suggesting this gene is a part of, or closely linked to, this *Ms* locus. This fertile *Ms* locus has been designated as *Ms45* and the Ac male-sterile allele designated as *ms45-m1::Ac*.

To confirm the phenotype for the *ms45* mutation, F_2_ progenies segregating for the *ms45-m1::Ac* allele were screened for Ac reversion and subsequent male fertility. One male-fertile F_2_ family containing a putative revertant *Ms45* allele, designated *Ms45’+7422*, was selected for further characterization. A 3’ portion of the *Ms45* cDNA was used to probe a *Pvu*II-digested genomic DNA blot of this family. This probe did not detect the presence of an Ac element, but it did reveal a polymorphism between the wild-type *Ms45* parental allele (3.8 kb-fragment) and the *ms45-m1::Ac* parental allele (3.4‑kb fragment).

To confirm the revertant, the F_2_ individuals were digested with *Pst*I and hybridized with a *Pst*I fragment derived from the *Ms45* cDNA clone. The *Ms45* wild-type parent was homozygous for an approximately 1.3-kb fragment. The *ms45-m1::Ac* parent was homozygous for a larger 6.0-kb *Pst*I fragment resulting from a 4.5-kb Ac insertion. All of the *Ms45’+7422* family F_2_ individuals were homozygous for the 1.3-kb fragment. The *Ms45’+7422* F_2_ individuals were shown to be derived from the *ms45-m1::Ac* parent but no longer contained an Ac element. This indicated that the transposon had excised from the *Ms45* gene and that genetic function had been restored, resulting in fertility.

Subsequent sequence analysis of the cDNA clone enabled design of PCR primers that bordered the Ac insertion site. These primers were used to amplify and sequence the revertant locus from homozygous *ms45-m1::Ac* derived plants. The *Ms45’+7422* allele was found to contain a 6 bp duplication at the Ac insertion site. This footprint duplicates two amino acids in the deduced protein sequence but leaves the translational reading frame of *Ms45* intact. Thus, genetic and molecular data confirm the excision of Ac from the *Ms45* gene.

A second excision event was found while genotyping a segregating *ms45-m1::Ac* F_2_ family. One male-sterile plant was genotyped as being heterozygous, containing both 1.3-kb and 6-kb *Pst*I fragments, rather than being homozygous for the 6.0-kb *Pst*I fragment as expected. PCR was used to amplify across the Ac insertion site, and the resultant product was sequenced. An 8 bp footprint was detected in this event, resulting in a frameshift in the translation of the gene and destroying *Ms45* gene function. This excision allele was designated as *ms45’-9301*. It represents a stable mutation of the *Ms45* locus and results in male-sterile plants when homozygous recessive.
